# Barriers and drivers to service delivery in global mental health projects

**DOI:** 10.1186/s13033-020-00427-x

**Published:** 2021-01-24

**Authors:** Onaiza Qureshi, Tarik Endale, Grace Ryan, Georgina Miguel-Esponda, Srividya N. Iyer, Julian Eaton, Mary De Silva, Jill Murphy

**Affiliations:** 1 Global Mental Health, London School of Hygiene and Tropical Medicine, Keppel St, Bloomsbury, London, WC1E 7HT UK; 2 Department of Counseling and Clinical Psychology, Teachers College, Colombia University, 525 W 120th St, New York, NY 10027 USA; 3 Centre for Global Mental Health, Department of Population Health, London School of Hygiene and Tropical Medicine, Keppel St, Bloomsbury, London, WC1E 7HT UK; 4 Institute of Psychiatry, Psychology and Neuroscience, King’s College London, 16 De Crespigny Park, London, SE5 8AF UK; 5 Department of Psychiatry, McGill University, 845 Sherbrooke St W, Montreal, QC H3A 0G4 Canada; 6 Douglas Research Centre, 6875 Boulevard LaSalle, Montreal, QC H4H 1R3 Canada; 7 CBM UK, 8 Oakington Business Park, Dry Drayton Rd, Oakington, CB24 3DQ UK; 8 Health of Population Health, Wellcome Trust, 215 Euston Rd, Bloomsbury, London, NW1 2BE UK; 9 Department of Psychiatry, Faculty of Medicine, University of British Columbia, 2255 Westbrook Mall, Vancouver, BC V6T 2A1 Canada

**Keywords:** Global mental health, service delivery, implementation factors, barriers, drivers

## Abstract

**Background:**

Research in global mental health (GMH) has previously documented how contextual factors like political instability, poverty and poorly-funded health infrastructure continue to compromise effective and equitable mental health service delivery. There is a need to develop more feasible and evidence-based solutions through implementation research. This paper, one in a series pertaining to implementation in GMH projects worldwide, focuses on implementation factors influencing mental health service delivery.

**Methods:**

This is a qualitative study carried out as part of a Theory of Change-driven evaluation of Grand Challenges Canada’s (GCC’s) Global Mental Health portfolio. Purposive sampling was used to recruit twenty-nine GCC grantees for interviews. A semi-structured interview schedule was used to guide the interviews which were recorded and subsequently transcribed. Transcripts were double-coded and analyzed in NVivo 11 using framework analysis. This paper reports results related to detection and treatment of mental illness, mental health promotion and prevention of mental illness.

**Results:**

Key barriers included: lack of appropriate human resources and expertise for service delivery; lack of culturally appropriate screening tools and interventions; and difficulties integrating services with the existing mental health system. Formative research was a key driver facilitating the cultural adaptation of mental health detection, treatment, promotion and preventative approaches. Recruiting local providers and utilizing mHealth for improving screening, monitoring and data management were also found to be successful approaches in reducing workforce burden, improving sustainability, mental health literacy, participant engagement and uptake.

**Conclusions:**

The study identifies a number of key barriers to and drivers of successful service delivery from the perspective of grantees implementing GMH projects. Findings highlight several opportunities to mitigate common challenges, providing recommendations for strengthening systems- and project-level approaches for delivering mental health services. Further, more inclusive research is required to inform guidance around service delivery for successful implementation, better utilization of funding and improving mental health outcomes among vulnerable populations in low-resource settings.

## Background

In the context of global mental health (GMH), “service delivery” refers to a structured set of implementation activities or interventions for mental health promotion, prevention, detection, treatment and support taking place on multiple delivery platforms [1]. Depending on the severity of the condition, the frequency of need, contextual considerations and human resource requirements and costs, service delivery can take place in different settings. These may include community-based settings such as homes, social welfare centres, local clinics, and schools; more general healthcare or specialist settings, mainly hospitals; or through projects with a preventative or mental health promotion approach [2]. The most common types of providers involved in the delivery of mental health services include specialists such as mental health practitioners and nurses, and non-specialists such as general practitioners and nurses, lay health workers, families and other community members [3].

A commonly reported barrier in the literature on GMH implementation is the shortage and unequal distribution of human resources for providing mental health services, which is more pronounced in low- and middle-income countries (LMICs) [3–6]. Mental health service delivery is also severely limited in most LMICs by fragmented health systems, ineffective referral pathways, lack of effective leadership, and inadequate financing mechanisms [7]. Researchers have recommended the decentralization of mental health services, provision of integrated and holistic care within primary health care and communities, and the strengthening of health systems [6, 7] to redress key barriers to GMH implementation. Ensuring the appropriateness of mental health interventions through formative research to understand and incorporate culturally and contextually appropriate approaches to mental health, well-being and recovery have also been identified as important considerations for successful mental health service delivery [8–10].

For over more than a decade, there has been a concerted effort to document and map the implementation processes necessary for the effective delivery of mental health projects in LMIC settings. This paper is one of a four-part series providing an in-depth analysis of the key barriers and drivers of implementation described by recipients of Grand Challenges Canada (GCC) Global Mental Health grants in the years 2012–2016 [11]. Given global under-investment and lack of implementation research in mental health [12], a thorough examination of the barriers and drivers to successful implementation is critical for more effective and equitable use and allocation of existing resources. Evidence-based recommendations for successful implementation can help strengthen the procedural and systemic building blocks required for promoting more sustainable mental health service delivery.

## Methods

### Aims

This paper describes results of a qualitative analysis of barriers and drivers to the implementation of service delivery in projects across GCC’s GMH funding portfolio [13, 14, 15]. For the purpose of this study, “barriers” and “drivers” refer to key factors that either positively or negatively affected a project’s ability to deliver an intervention as described in its initial funding proposal. Based on the results of a quantitative analysis of GCC-funded project outcomes using a portfolio-level Theory of Change (ToC) approach [16], six key themes emerged as important to implementation success: (1) Stakeholder engagement; (2) Training providers; (3) Supervision of providers; (4); Detection of mental illness; (5) Treatment of mental illness, and; (6) Mental Health promotion and prevention of mental illness. This paper presents findings for the service delivery cluster, which comprises themes 4–6 (detection, treatment, promotion, and prevention).

### Data collection

**Fig. 1:**
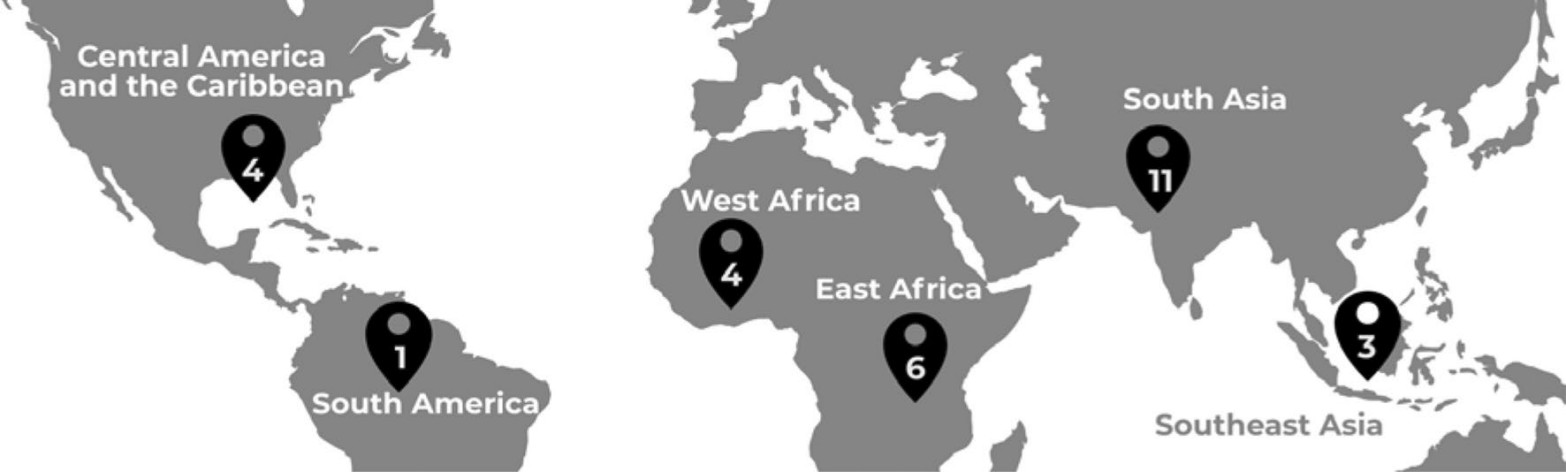
Global distribution of projects included in the analysis

This study took place between June 2014 and May 2017 through four rounds of data collection. We used purposive sampling to recruit participants who are current or former GCC Global Mental Health grantees. Participants were approached during two GCC community meetings in the United States and United Kingdom for face-to-face interviews with members of the study team. Recruitment continued after the meetings with standardized participation templates, information sheets, and consent forms sent by email. Interviews were conducted in-person or by Skype. 29 grantees participated in interviews, representing grantee leads or co-investigators in GCC-funded projects in Latin America and the Caribbean (n = 4), South America (n = 1), West Africa (n = 4), East Africa (n = 6), South Asia (n = 11) and Southeast Asia (n = 3) (Fig. 1).

Study participants represented projects targeting a variety of mental health and substance use disorders [15], and were at different stages in their funding cycle with GCC at the time of interview. Members of the research team conducted the interviews in English and recorded them with the consent of participants. A semi-structured interview guide was developed to explore each step on a collective Theory of Change (ToC) map representing projects in GCC’s Global Mental Health funding portfolio, as described elsewhere in this volume [15]. Grantees were asked to choose which steps they felt were the most important to discuss in relation to their projects, and to describe what helped or hindered their success in completing this step. Interviews ranged from approximately 30–60 min in length. Data saturation was defined as “the point where no new information was added to the codebook” [17] and was achieved after four rounds of data collection. Ethics approval was granted by the London School of Hygiene and Tropical Medicine’s Research Ethics Committee (#7746 and #9945).

### Data analysis

**Fig. 2:**
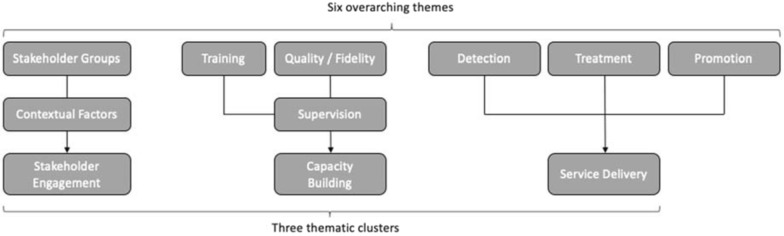
Breakdown of thematic clusters

 Audio recordings were transcribed for analysis using a framework approach, which has been widely used in health policy research to identify barriers and drivers [18]. Three members of the research team (JM, OQ, TE) conducted coding using NVivo 11 software, with JM coding the full data set and OQ and TE each coding sixteen interviews. Following immersion in the data, JM developed an initial codebook, which was discussed and refined by the coding team. We coded three interviews using the refined codebook and ran a coding comparison in NVivo 11. The team then discussed areas of divergence, further refined the codebook, applied it to two additional interviews, and developed a finalized version that was utilised in the remaining interviews. Based on previous research and emerging results during the analysis process, the research team grouped the six key themes into three thematic clusters as defined and shown in Table 1 and Fig. 2: (1) Stakeholder Engagement; (2) Capacity building, and; (3) Service delivery.

**Table 1:** Definition of sub-themes under ‘Service Delivery’

Service Delivery Sub-themes	Definition
Mental health promotion and awareness	Refers to any interventions or activities that were conducted with the purpose of promoting improved awareness and understanding about mental illness, including among patients, healthcare workers, community members or policy makers.
Detection of mental illness	Refers to structured steps or activities that are taken with the purpose of detecting mental health symptomology or disorders among patients or community members.
Treatment of mental illness	Refers to interventions that are provided to people diagnosed with mental disorders (or their relatives) and which are aimed at alleviating psychiatric symptomology, supporting people to self-manage their symptoms or promoting wellbeing.

Following the coding process, we used the codebook to create an analytic framework that allowed for the identification of emerging themes for each of the three clusters. The framework was populated separately and discussed, using an iterative process, to arrive at consensus about predominant themes.

This paper presents findings for the Service Delivery cluster, with results from the other two clusters published elsewhere in this volume [13, 14].

## Results

The interview participants included in our sample were either program leads or co-investigators leading on a global mental health project in their respective settings. General characteristics of the projects that were implemented by the 29 interviewees are outlined in Table 2. While many projects targeted multiple disorders and population groups, a large majority of the projects focussed on alleviating common mental health disorders and behavioural and emotional disorders in their settings. A wide variety of groups were targeted by the projects, with a majority focusing their intervention towards children and adolescents (from 1 month to 14 years) as well as young and older adults (from 15 to 60 years). Around 34% of the projects also targeted vulnerable groups such as those affected by natural disasters or conflict. Capacity-building (79%), detection, treatment, care and rehabilitation (76%) were the most commonly incorporated elements to the interventions conducted by the projects. While fewer projects incorporated elements of stakeholder engagement in their projects, findings from the stakeholder engagement paper in this series highlighted how essential and cross-cutting this component was in driving success for a variety of project outcomes [14].

**Table 2:** General characteristics of global mental health projects being implemented by interview participants

Project Characteristics	Qualitative study (n = 29) N (%)
Target disorder	
Common mental disorders	16 (55)
Behavioural and emotional disorders	13 (45)
Trauma and PTSD	7 (24)
Suicide and self-harm	7 (24)
Developmental disorders	7 (24)
Severe mental disorders	6 (21)
Epilepsy and seizures	5 (17)
Alcohol and substance use disorders	5 (17)
Dementia	2 (7)
All	3 (10)
Target population group	
Children and adolescents [1 month – 14 years]	14 (48)
Young and old adults [15–60 years]	13 (45)
Vulnerable groups [e.g. conflict afflicted populations]	10 (34)
Women	9 (31)
Elderly [over 60 years]	6 (21)
Newborns [under 1 month]	2 (7)
General population [all ages]	12 (41)
Region	
South Asia	11 (38)
Africa	10 (35)
Central America and the Caribbean	4 (14)
South East Asia	3 (10)
South America	1 (3)
Innovation components	
Capacity building	23 (79)
Detection, treatment, care and rehabilitation	22 (76)
Promotion and awareness	18 (62)
Stakeholder engagement	12 (41)

### Mental Health Promotion and Awareness

Although distinct in principle, mental health promotion and awareness-raising activities often overlapped in practice. These were combined into a single category described by interview participants as comprising activities or interventions conducted to improve the knowledge, awareness, and understanding of mental health and illness among patients, communities, policymakers and other stakeholders in their projects.

### Barriers

Awareness-raising activities to increase community knowledge and demand for care were challenging to implement in settings where services were limited or not easily accessible. Participants reflected on how this might impact their ability to deliver on the expectations of those requiring support and referral services:

*Identifying people with [mental health conditions] is not the end, it’s just the beginning. Where do you refer them? We have to refer them to the clinic. Who is going to treat them? Is the health system ready to address [the need for services]? Because what I’m seeing here, in most of the projects, of course we’re trying to raise awareness and identify people with mental health problems, but the system is not really prepared to address the issues.* (Participant 19)

### Drivers

Drivers for the successful implementation of mental health promotion included recruiting providers who were based in the communities where they worked. Grantees felt this would also be a more sustainable approach to improving mental health awareness long after project activities ended, as these providers would be able to continue to support their communities. Motivation was identified as another driver for the continuation of mental health promotion, as providers felt empowered by the positive impact of their work through their interactions with beneficiaries.

Reframing mental health in culturally appropriate terms was discussed as a way to prevent stigma through awareness-related activities. Participants pressed on the importance of promoting mental health in ways that were deemed locally acceptable. In the following quote, a participant reveals how they side-stepped the problem of stigma by framing their promotion activities as ‘resiliency services’:

*--, we’re not selling the services as mental health services. We’re selling them as wellness services and resiliency services as coping with chronic health disease [sic]. You know because there is stigma around mental health in the country.* (Participant 9)

### Detection of mental Illness

Participants described this theme as comprising activities implemented to detect psychiatric symptoms or diagnose mental disorders among patients or community members. The most common mechanisms for detection mentioned in interviews were assessment tools administered by trained formal or informal providers, using either paper forms or mHealth applications.

### Barriers

A major challenge in the implementation of mental health detection was the lack of appropriate human resources. As described further by Endale et al. [13] in an accompanying paper, this was related to barriers in retaining providers, stigma associated with working in mental health services, and the absence of specialists needed to conduct diagnostic evaluations.

Challenges related to the lack of understanding of cultural variations and idioms of distress also led to concerns over identifying valid screening tools for the target population. In one interview, a participant explained how they had to expand their efforts at detection from health centres to the general population, as mental health literacy is low [19] and some diagnoses do not fit with local approaches to mental health:

*So because help-seeking is really low and because awareness of primary care providers is really low, we couldn’t [sic] we asked them to screen everybody who came to the primary care centres instead of people who were symptomatic or people who would like come in and say, ‘Hey I’m depressed,’ because that doesn’t happen in [country name].* (Participant 12)

Some projects also reported having insufficient research expertise within their team to conduct validity and reliability analyses of their screening tools. Incorrect cut-offs for mental health diagnoses in tools can lead to either false positives or negatives. Moreover, participants outlined how it was especially difficult to identify locally appropriate tools for less common conditions (e.g., behavioural disorders):

*… for the scale-up, that became a problem, because people with mild to moderate depression, people who scored [below the cut-off] needed to utilize the intervention as well. And in some cases we had people who even scored [quite a bit below the cut-off score] who had genuine problems and needed to get–, to receive services. Which obviously has a lot to do with the sensitivity and specificity of the screening tool.* (Participant 22)

### Drivers

One participant reported that incentive schemes and team-building activities were useful in motivating frontline providers. Concerted efforts to incentivize collaborative working were found to mitigate professional issues (e.g. negative competition or disagreements) and achieve higher screening rates.

Participants also stressed the need for an integrated approach to adapt and improve detection, calling attention to the need to include key beneficiaries in both the initial design and the later implementation stages. Examples of approaches included integrating screening into routine community detection efforts for other health conditions (e.g. Interactive voice response system for maternal health) and making the screening forms and questions easily accessible to providers in health settings, such as by laminating questions and framing them on clinic walls.

Using technology to make detection easier for providers emerged as another key driver. Participants highlighted mobile health (mHealth) applications as a way to make screening easier, faster and less vulnerable to human error. The role of technology in managing data more effectively was also mentioned, particularly in regards to promoting a more efficient monitoring and evaluation system compared to using paper forms and filing systems:

*... developing the app was instrumental in being able to screen this number of people, because the data management, data cleaning all of that was just all electronic and in real-time. So I think that really helped.* (Participant 7)

### Treatment of Mental Illness

The theme of treatment referred to participants’ self-reported experiences in implementing interventions provided to people diagnosed with mental disorders (or their relatives) during the timeframe of their grants. These interventions were aimed at alleviating psychiatric symptomology, supporting people to self-manage their symptoms and promoting wellbeing.

### Barriers

Several participants highlighted major barriers related to the feasibility of implementing ‘novel’ treatment modalities or intervention designs (i.e. those that have been recently recommended in global literature) in their settings. While there may be international pressure to adopt new interventions, participants argued that it might not be realistic to implement in specific contexts. For settings struggling with limited resources, these challenges can impede efforts to scale up or sustain project activities. Others found it challenging to find the right balance between delivering interventions with fidelity and making room for adaptations to improve local appropriateness. Projects that struggled to implement ideal treatment protocols found inconsistencies between what global literature purported and their own treatment approach outcomes. According to two interviewees, the differences occurred due to the interventions being ‘Western’ in their approach, making them difficult to adapt to vastly different belief systems:

*There’s so much of these therapies that are really Western in thinking, right? And they’re not, it’s not that they’re contradictory to the culture but they’re a little bit more progressive than the ordinary thinking.* (Participant 9)

Some participants found it difficult to integrate mental health into health care settings. They ascribed a number of challenges to the inefficient care pathways in which they were working, including finding appropriate referrals for patients, the lack of qualified mental health professionals and services (particularly in rural settings), and inefficient existing mental health services. These inefficient care pathways added to the burden on their project workforce and limited the scope of their interventions.

*..we learn that there are [sic] lack of services in both early identification and also intervention for the [participants] with [mental health conditions]. And because the services there, they only focus [sic] in the big cities.* (Participant 26)

Some participants shared challenges they faced as a result of underestimating the level of demand for their services. In most cases, the level of demand did not seem to match the epidemiological evidence for uptake or the experiences of previous studies conducted in similar settings. Unexpectedly high levels of demand could over-burden the existing workforce, exceeding the limited capacity of the service providers and generating more pressure for additional funding to keep services running beyond the timeframe of the grant:

*They have expressed a great need for the program and had [sic] expressed to us that we should continue the program, which is making life difficult for us, because now we are trying to find how we can generate funds to keep the program going…* (Participant 17)

Participants also identified a series of common challenges (summarised in Table 3) related to the technical aspects of implementing treatment interventions for mental disorders.

**Table 3:** Summary of additional findings on the technical challenges faced by interviewees in the delivery of mental health interventions

Challenges		Quote
Technology		
Limited financial and human resources and expertise to troubleshoot technical issues Service disruptions due to infrastructure issues, e.g. poor network service in rural areas Some interventions required service users and their caretakers to use mHealth. This required technical expertise that they may not possess		*‘..there could be a very fancy technological app that could capture everything […] but that would cost so much, that would need a lot of human expertise, that – that (sic) might not be able to scale it up in for example other resource-poor settings’* (Participant 6)
Logistical difficulties		
Barriers to implementation and travel due to challenging terrain and geography, e.g. rural or mountainous areas Unanticipated delays due to the extended periods of time needed to train, deliver interventions and motivate stakeholders to participate Difficulties in cooperating with bureaucratic and conventional systems, e.g. a disruption in service delivery if administrative processes are not adhered to correctly and drug procurement problems		*‘And some of them, our service users were shepherds which, you know shepherds they come down from the mountains for the services, and then they go back. So it’s very difficult to actually reach them’* (Participant 25) *‘..making sure that there is a supply of drugs is very important. Quite a lot of time is spent with the government trying to see how they can increase that flow’* (Participant 4)
Financial limitations		
Implementing in populations with financial limitations kept their stakeholders from participating when they required money for travel or additional tests		*‘..for some of the participants who were living far from the centers it was a challenge to have them come to the sessions because some of them don’t move around a lot’* (Participant 10)

### Drivers

The local adaptation of materials required to implement the interventions was found to be essential for successful implementation and participant uptake. Interviewees identified multi-modal methods that improved project acceptability to stakeholders. The methods identified include [1] engaging with service users in the development stages, [2] tailoring the intervention to participant needs, [3] maintaining the dignity of services users, [4] integrating within existing cultural practices and [5] building in strong systems of support that were empowering and beneficial to end-users beyond the scope of the project:

*I think part of that is kind of recuperating cultural practices that may be in part disintegrating due to the change but also to create support networks between people* (Participant 18)

Stakeholder-driven promotion is another driver that enhanced implementation success through improved engagement within the targeted populations. As described further by Murphy et al. [14] in an accompanying paper, integrating positive treatment outcomes into dissemination efforts drastically improved chances of the intervention’s success in increasing participant acceptance, adherence and the uptake of services.

*People can see, you know, the quick outcomes of this intervention. But this has been the role of advertising. Successful cases played the most important role* (Participant 20)

Closely linked to the sub-theme of service uptake was using the increased demand for services to garner buy-in from a larger pool of stakeholders, including policymakers, in order to build the intervention delivery into a sustainable and scalable model. One interviewee also explained that this might sometimes require being flexible and deviating from the intervention protocol.

Aligning service delivery within existing care pathways was also perceived to have a significant impact on implementation success. Interviewees discussed how training and using lay workers that were already connected to local services to deliver their interventions could address human resource gaps, in addition to allowing for referrals and the provision of support to end-users. Because of these learnings, the need for integrating mental healthcare into locally applicable and socially supportive referral networks was identified as an important driver for greater reach, impact and success of service delivery.

*So for us then… it made sense to go with the social franchising model and family care […] kind of combines the best of all those social franchising operations around the country. So they have networks everywhere* (Participant 9)

Involving family members in treatment activities for benefiaries played a significant role in implementation success. Involving families helped to reduce the burden on limited human resources, empowered families and gave them the skills and confidence to better care for their relatives. Involving family members also encouraged the building of sustainable support systems between different participating family units within the same areas.

*..So we are shifting the task even from the [name of lay health workers] to the families. And that’s a sustainable and scalable model* (Participant 6)

Only one participant flagged the engagement of family members as a barrier, and this may be specific to service delivery approaches for substance use disorders. These considerations are important to note for disorders that are comparatively more socially stigmatizing than other mental health conditions in certain settings.

*…and it also disrupts the session, in the sense that the family members might not talk about a lot of things that might come up because the man is sitting in [at the] back, saying things which he might not like [sic] might mean* (Participant 17)

Health technology solutions, while challenging to maintain, were also identified as an important driver, providing flexibility to implementers in adapting their interventions, sharing lessons learned, and utilizing more efficient systems of monitoring and evaluating projects. For some, mHealth solutions supported a larger coverage of interventions and detection efforts, and provided projects with a platform for learning how to improve their services:

*We moved to smartphones, and we get – that has given us so much more flexibility in terms of, you know, enhancing our interventions, making like applications, getting all of the information to them for health providers to use for their own learning and for the learning of the community when they go and visit those people […] So I think that there have been these facilitators mainly.* (Participant 24)

## Discussion

It is clear from the interviews conducted for this study that there are common drivers that implementers have harnessed to improve their chances of success across a diverse portfolio of global mental health projects. Similarly, there are common barriers to be overcome. Despite the often complementary experiences of grantees in addressing these implementation factors, participants often reported feeling isolated in their efforts to problem-solve, reinforcing the need to harness and share learning across projects. In this paper, we have captured key findings related to three areas of mental health service delivery: promotion and awareness-raising, detection and treatment.

### Promotion and awareness

Integrating awareness-raising and other promotional efforts within mental health service delivery are key strategies to increase uptake in treatment participation and help-seeking for mental health services and to mitigate the harmful consequences of stigma. Yet, participants raised concerns around the implications of promotional efforts that could generate more demand for mental health care than a project has the capacity to address. Raising awareness in LMIC settings, where services for mental health are lacking and fragmented—and sometimes of poor quality—is an obvious ethical issue. To address this concern, project teams dedicated to promotion and awareness-raising should consider the current capacity of their mental health systems, and align their work with existing services. System-strengthening and integrating promotional efforts within existing resources in the setting (e.g. recruiting lay health workers who already worked within the same communities) increased the long-term likelihood for sustaining mental health services and creating more supportive networks for service users in their communities [20].

The importance of culturally-appropriate mechanisms of awareness-raising was highlighted by the concern that awareness-raising messages may inadvertently create more stigma or propogate existing prejudices around mental health if they are not adapted to the targeted population. Successful approaches for mental health awareness-raising included recruiting providers from the same community (e.g. peer or community health workers) and focusing on integrating promotional components within existing systems of belief [21], e.g., rebranding services as ‘wellness’ approaches. Similar practices should be adopted to promote the uptake of services in a safer way for targeted populations to access care without feeling stigmatized. These findings support existing evidence that recruiting providers from similar communities as beneficiaries, to deliver interventions, can have a positive effect on stigma reduction [22]; and that projects should develop more contextually and culturally appropriate pathways and messaging for their promotional efforts [23].

### Detection of mental illness

The challenges and implications of delivering services within the context of a poorly resourced workforce were major concerns emerging from the interviews. The primary barrier identified was the lack of mental health specialists for guiding the design and implementation of effective mental health detection efforts. The dearth of research expertise landed the burden of developing effective tools for detection on local implementers who lacked the clinical knowledge, theoretical basis and necessary expertise in carrying out feasibility and validity studies. While some projects partnered with academic institutions in high-income countries for this purpose, building local research skills and expertise is recommended as a more sustainable approach to addressing this barrier. Collaborative partnerships with mutual learning, support and supervision can help bridge the ‘research expert gap’ and redress inequal power dynamics between research instititions from lower vs. higher-resource settings [24].

The use of standardized tools for detection was found to be both a driver and a challange in service delivery. The availability of internationally validated tools aided in implementing a monitoring and evaluation plan without the added burden of validity testing. However, projects that were unable to invest in the assessment of instruments were more likely to select standardized tools with limited evidence of cross-cultural validity. Using tools with limited evidence of validity in local settings can lead to category fallacy [19] and an inaccurate assignment of cut-off scores for specific populations, given the diversity of socio-economic determinants for mental illness in different settings and the cultural differences in expressions or manifestations of distress [25]. One participant reported that incorrect cut-off scores for the selected instrument resulted in a larger pool of recipients for care than previously budgeted for and added additional strains on their workforce. While effective and brief screening tools are available [26] and necessary for project implementation in low-income settings, there is a need to assess their cross-cultural applicability to account for local idioms and definitions of distress [27] and mitigate the risk of labelling and stigmatization [28].

Study findings highlighted the utilization of mHealth technologies as an essential driver in service delivery, to aid monitoring and evaluation and detection for mental health projects. The uptake of mHealth technology is supported by a rapid increase in phone ownership in LMICs [29], and the evidence of its effectiveness in mental health projects [30, 31]. Although opportunities for integrating mHealth into routine case finding efforts for mental health are abundant, care should be taken to ensure that mHealth technologies are feasible in the context of the targeted population, and there is sufficient information technology and data management infrastructure available to address any potential issues that may arise. As the evidence in this area is still limited, implementers should evaluate the impact of mHealth on project monitoring, successful identification of common and severe mental illness, and improving the efficacy and quantity of screening and case-finding.

### Treatment of mental illness

Challenges around the acceptability and feasibility of mental health interventions for both providers and recipients came through as a key finding of this study. Competing for international funding sources may put pressure on local implementing agencies to adopt ‘novel’ approaches that they struggle to implement in their setting due to varying contextual factors: “..And you know their [global funder] funding approach is actually occasionally to fund quite radical ideas, because it’s an interesting theory to prove or disprove, but the implementation is so hard that you’re never going to actually get to proving or disproving that theory” (Participant 19). A long-standing criticism of the global mental health agenda is that it promotes ‘Western’ and/or ‘bio-medical’ approaches for mental healthcare in LMICs [32] and there is universal agreement on the need to culturally adapt interventions to local settings and targeted populations [25]. However, it is often left to project teams to find the right balance of introducing locally appropriate adaptations without significantly affecting the fidelity of a particular intervention. The additional responsibility of delivering interventions in line with their protocol while ensuring that they are acceptable to participants and amenable to change is highlighted in recent literature [33]. Investing in feasibility studies and formative research [34] prior to the adoption of new practices is beneficial to the overall sustainability of the projects and seen as one way to address this challenge. The overall acceptability and uptake of interventions is supported by building in a level of flexibility within the protocol and engaging key beneficiaries from the initial stages of service delivery design and implementation.

Determining the level of service demand an intervention may have within targeted populations is addressed through formative research methods, including situational analyses and needs assessments [9]. Despite taking these measures, over or underestimating the level of demand for services was a commonly faced barrier to successful delivery for the projects included in the study. Implications of increased or low demand put projects at risk of fund mismanagement due to the misallocation of human resources or a potential increase in the workload of health workers. While implementers must utilize the existing resources available to them, investing in interventions that bolster their health system’s ability to measure, monitor, and strengthen care pathways for mental health [35], will over time create a more accurate measure of service need and utilization. Moreover, focusing on formative research to understand the knowledge, attitudes, and practices behind mental health and integrate traditional pathways for demand can facilitate the development of a model that is acceptable, culturally appropriate, and more likely to be sustained at scale.

The service delivery agents utilized within projects were typically dictated by the type of treatment approach, the mental health condition being targeted and the chosen delivery platform, i.e. community-based setting or primary-care. There was consensus among the grantees who engaged peers (described by interviewees as persons who had lived experience of mental health conditions or shared experiences of caring for people with mental health through their work, in their communities or families) that this was beneficial for service delivery. Using participatory approaches to harness peer support in project delivery can improve the level of engagement and participation with beneficiaries. While there is limited evidence for the mental health outcomes [36, 37] and sustainability of peer support and family member involvement in service delivery, findings from this qualitative analysis reinforce positive findings from the existing literature in this area [38]. Given the gaps in the existing evidence, more research is needed before conclusively recommending mechanisms for integrating more comprehensive peer support components in mental health care delivery.

Projects invested in creative approaches to adapt services for their target population’s socio-cultural environment, norms and language, and promoted this as a successful driver of their intervention delivery. Murphy J et al. 2020 also reiterate how local adaptation enhances participation, creates more supportive systems of care for communities with fragmented healthcare pathways for treatment, and reduces the likelihood of remission [39]. Utilizing successful examples of interventions to standardize methods and develop normative guidelines for the appropriate adaptation of intervention delivery components has the potential to facilitate and evaluate the impact of this driver in future mental health service delivery projects. Additional examples of factors facilitating successful service delivery mechanisms by the projects interviewed are outlined in Table 4.

**Table 4:** Approaches and mechanisms for successful service delivery

Themes	Key recommendations
Fragmented and poor care pathways	Integrate awareness-raising efforts as part of routine mental health service delivery
Contextual and cultural adaptability	Invest in system strengthening to improve coordination between different levels of providers
	Invest in participatory formative research to better understand context and need
Lack of mental health human resources	Engage with key stakeholders from the initial stages of project design through to delivery to increase investment and uptake
	Utilize existing community resources, stepped-care approaches and task-sharing with non-specialist providers
Measurement and detection	Allocate funding towards developing measures that are easily adapted to different (low-income) settings
	Build capacity within local implementing agencies to carry out formative research on the validity and reliability of screening tools
Fidelity in the intervention protocol	Invest in feasibility and appropriateness studies prior to implementation
	Build an iterative design and feedback into delivery mechanisms to integrate key learnings for continued contextual adaptation

### Limitations

The interview respondents were not always direct implementers of their projects and so were only able to provide a high-level picture of implementation challenges and barriers. A more accurate and technical viewpoint would require investigation with the local team of providers. Many interviewees were also at different stages of their project implementation, and this may have affected the types of challenges and drivers they reported. However, this was also a strength in that it allowed us to glean insights about barriers and drivers to implementation across a diverse portfolio of projects at different stages.

While the semi-structured format of the interview process was beneficial in that interviewees chose to discuss the themes and topics that were most relevant to them, it resulted in discrepancies between the types and amounts of information reported regarding certain approaches, which limits the generalizability of the findings. Only a few of the projects included in this study focused exclusively on discrete promotional activities, while others included these activities as an added component in the overall project goals. It is possible that self-selection of the themes by participants during the interview process could have created a bias in selecting mental health treatment and detection as the more critical component of their mental health project.

There is also potential for bias here as GCC grantees were aware that the research was being conducted by members of the Mental Health Innovation Network which at that time was a GCC-funded knowledge exchange and support platform for the innovations developed through the portfolio. However, grantees were assured that confidentiality would be maintained and that no identifying features of their project would be shared with the funder or attributed to them in publications or other materials resulting from the study. Moreover, records were not kept for mode of interview (e.g. in-person versus by Skype) or refusal to participate, which may have given further insight into the type of participants that were ultimately enrolled in the study.

## Conclusions

This study identified the drivers of and challenges to the implementation of mental health service delivery in a portfolio of projects from Grand Challenges Canada’s Global Mental Health funding stream. The findings from this study describe how important it is to utilize existing human resources for feasible, acceptable and sustainable mental health service delivery. It also highlights how investing in formative research and system strengthening in the design stage and in all aspects of project delivery is crucial for addressing challenges related to cultural appropriateness, capacity, monitoring and evaluation, detection and identification efforts, and ensuring functioning care pathways for people in need of mental health care. In response to a gap in the measurable impact of mental health promotion and prevention, funders need to invest in more focused streams for implementing and evaluating awareness and preventive components of mental health projects to promote this area as a priority and measure its impact on inter-sectoral benefits to health system strengthening and building political will.

The study highlights important key lessons to contribute to our understanding of ‘what works’ in service delivery for global mental health projects in LMICs. Furthermore, it provides insights on how to anticipate and mitigate common barriers to successful implementation and, in this light, has implications for local project implementers, funding agencies and researchers invested in promoting successful global mental health project delivery. As noted in other papers within this series, the need to meaningfully engage with relevant key stakeholders from the earliest stages of project development and to build capacity including through sustainable supervision of recruited workers are essential for the successful delivery of mental health projects.

## Data Availability

The quantitative data that support the findings of this study are available from GCC, but restrictions apply to the availability of these data, which were used under license for the current study, and so are not publicly available. These data are however available from the authors upon reasonable request and with permission of GCC. The qualitative data generated during the current study are not publicly available due to the sensitivity of discussions surrounding the performance of grantees’ projects but are available from the corresponding author upon reasonable request.

## Abbreviations

GMH: Global Mental Health
GCC: Grand Challenges Canada
mHealth: Mobile Health
LMICs: Low- and Middle- Income Countries
IT: Information Technology
MHIN: Mental Health Innovation Network
